# Validation of Biomarkers and Immunotherapy With Crohn's Disease Using WGCNA and Two-Sample Mendelian Randomization Study

**DOI:** 10.1155/grp/8194480

**Published:** 2025-07-01

**Authors:** Cong Hu, Shuxiong Nong, Chenang Liu, Yongfeng Chen, Chilin Liao, Meng Wu

**Affiliations:** ^1^Department of Ultrasound, Zhongnan Hospital of Wuhan University, Wuhan, Hubei, China; ^2^Department of Cardiology, Baise People's Hospital, Affiliated Southwest Hospital of Youjiang Medical University for Nationalities, Baise, Guangxi, China; ^3^Teaching Office, Zhongnan Hospital of Wuhan University, Wuhan, Hubei, China; ^4^Social Medical Development Department, Zhongnan Hospital of Wuhan University, Wuhan, Hubei, China

## Abstract

**Objective:** Crohn's disease (CD) is a chronic systemic inflammatory disease that mainly affects the intestine, accompanied by extraintestinal symptoms and immune problems. The progression of the disease may cause permanent damage to the structure and function of the intestine. Due to unclear early symptoms and lack of precise detection methods, early diagnosis of CD is difficult. Many patients were diagnosis at late stage, which may lead to delayed treatment and increased risk of complications. Identifying hub genes related to CD and using them to predict CD is of great significance.

**Methods:** DEG and WGCNA were employed to identify key genes associated with CD and to detect modules significantly linked to the disease. GO and KEGG analyses were conducted to explore the functions of these identified genes. Additionally, MR method was utilized to assess the causal relationships between the most significant gene and CD.

**Results:** WCGNA identified 3240 differentially expressed genes, with the magenta module being the most significant among the nine clustered modules. The enrichment of GO and KEGG pathways indicates that the hub genes in the magenta module are related to the positive regulation of heme binding, tetrapyrrole binding, carboxylic acid binding, organic acid binding, IL-17 signaling pathway, and amoebiasis pathway. The Top 5 hub genes are CXCL1, LCN2, NOS2, S100A8, and DUOX2. Mendelian randomization analysis found a significant correlation between CXCL1 and CD.

**Conclusions:** The study screened five potential biomarker genes in CD patients using a bioinformatics approach and Mendelian randomization study. Our results provided insights into CXCL1, LCN2, NOS2, S100A8, and DUOX2 in CD and suggested that CXCL1 may potentially be the optimal biomarker that could be a relatively easy path to clinical translation.

## 1. Introduction

Crohn's Disease (CD) is a chronic inflammatory bowel disease (IBD) that can affect the entire digestive tract from the mouth to the anus. The symptoms are diverse, including abdominal pain, diarrhea, weight loss, fatigue, fever, and gastrointestinal bleeding [1]. The manifestations of CD can seriously disrupt the daily operations of affected individuals, reduce their work performance, cultivate a sense of isolation, and trigger psychological stress [2]. Furthermore, the presence of CD increases the risk of developing additional health complications which may worsen the patient's condition and even require surgical treatment.

Biomarkers serve as crucial indicators in CD patients, offering insights into the various biological activities and mechanisms occurring within the human body [3, 4]. The identification of biomarkers for CD is receiving increasing attention from researchers. The establishment of promising biomarkers for CD is of great importance, because it will provide personalized and precise treatment for patients, assess their condition, and predict prognosis [4]. In bowel disease research, the bioinformatics and microarray analysis has marked a significant progress. Recent investigations used the tools of microarray expression profiling on tissues from Crohn's patients, specifically from the ascending/descending colon and terminal ileum, to find hub genes related to the disease [5, 6]. WGCNA was used to analyze the intricate and complex network relationships and the potential molecular mechanisms [7]. WGCNA operates by forging a gene expression network, clustering genes that exhibit high correlation into modules of coexpression, thus showing the links between these modules and distinct sample traits [8].

Through single-cell transcriptomics technology, researchers are able to classify and analyze various cell types in the intestinal tissue at a high resolution. Studies have revealed the heterogeneity of macrophages and neutrophils in the intestines of patients with CD, identifying differences in the distribution and function of different macrophage subpopulations at inflammatory sites [9]. Moreover, multiple distinct cellular states and subpopulations of intestinal epithelial cells have been discovered, which exhibit diverse gene expression patterns in response to inflammatory stimuli. Single-cell techniques have uncovered complex signaling pathways between intestinal epithelial cells and immune cells, which play a significant role in the pathogenesis of CD. They have also revealed interactions between stromal cells and immune cells, which may influence inflammatory responses and tissue repair. Single-cell transcriptomics research has provided new clues for predicting the therapeutic response of patients with CD [10, 11]. By analyzing the cellular transcriptomic changes before and after anti-TNF treatment, it has been found that responders and nonresponders exhibit different gene expression patterns in monocytes/macrophages and fibroblasts. Spatial transcriptomics technology enables researchers to link the transcriptomic information of cells with their spatial locations within tissues. Studies utilizing spatial transcriptomics have analyzed the cellular composition and gene expression patterns in different regions of the intestines of patients with CD [9, 12]. It has been found that the intestinal tissues of CD patients harbor specific inflammatory microenvironments, where cellular interactions and signaling pathways are closely related to the occurrence and development of the disease.

Mendelian randomization (MR) uses instrumental variables (IVs) to infer causal relationships [13]. Using data from GWAS, offering a pathway to identify unbiased effects, eliminate confounding factors and reverse causal relationships, and improve results credibility. In this work, we identified differentially expressed genes (DEGs) advantages of this research compared to other studies. WGCNA was then utilized to recognize the most relevant modules for CD, which greatly narrowing the scope of genes to screen for. The causal relationship between CXCL1 and CD was explored through MR study.

## 2. Methods

### 2.1. Data Source

The Gene Expression Omnibus (GEO) was collection of datasets encompassing microarray data and other forms of genomics [14]. In the context of our research, we have successfully found microarray datasets associated with CD by conducting keywords search for “Crohn's disease” within the GEO database. The dataset GSE179285 (including 168 CD patients and 31 controls) was downloaded from GEO.

### 2.2. Data Normalization and DEGs

We first normalized the GSE179285 dataset using R statistical software (4.4.1). We conducted differential analysis using the “limma” package in R to compare gene expression differences between the CD group and the healthy control group. We set two filtering thresholds: *p* < 0.05 and an FC > 1.5. These thresholds help us screen for genes with statistically significant differences, while ensuring that these changes are also biologically meaningful. To visually demonstrate the expression patterns of these DEGs, we used the “pheatmap” and “ggplot2” packages to create heat maps and volcano maps.

### 2.3. WGCNA

A coexpressed gene network was constructed through a series of steps, including sample clustering analysis to identify and remove abnormal samples, and the use of a one-step network building function to establish a scale-free coexpressed gene network [13–15]. We used the R function “pickSoftThreshold” to calculate the soft threshold (*β*). Next, we transformed the adjacency matrix into a topological overlap matrix (TOM) and calculated the corresponding dissimilarity (1-TOM). This step helps us better understand the co expression relationships between genes. By utilizing hierarchical clustering and dynamic tree segmentation functions, we detected modules in the network that may represent specific biological processes or disease-related gene sets. By calculating gene significance (GS) and module membership (MM), we identified modules related to clinical attributes, which helps us further understand the molecular mechanisms of CD.

### 2.4. GO and KEGG Analysis

We conducted GO functional analysis and KEGG pathway enrichment analysis on the selected DEGs. These analyses help us understand the potential biological functions of these genes and the biological pathways they involve. We used the “clusterProfiler” package for these analyses, which provides a range of tools to evaluate the enrichment of gene sets [16]. We set the significance threshold to a *p* < 0.05 to ensure that the biological processes and pathways we identify are statistically significant.

### 2.5. PPI Construction and Hub Gene Identification

To further understand the functions and interactions of these DEGs, we constructed a protein–protein interaction (PPI) network using the STRING database. This database provides a large amount of PPI data, which helps us construct and analyze complex protein networks. We visualized the PPI network and identified important interacting genes in the network using Cytoscape software and CytoHubba plugin. These genes may play a central role in the pathogenesis of CD.

### 2.6. Nomogram Construction

Using the “rms” package, we constructed a column chart, which is a graphical tool that can help us predict the risk of CD based on feature genes. In the nomogram, we show the individual scores of different genes and the total score of all genes, which can be used to estimate the incidence rate of CD. To evaluate the specificity and accuracy of these genes in diagnosing CD, we used ROC curves. The area under the ROC curve (AUC) is an important indicator that can help us evaluate the performance of these genes in diagnosing CD.

### 2.7. Immune Cell Infiltration Analysis

To evaluate the abundance of infiltrating immune cells in the expression profile of CD related genes, we used the CIBERSORT algorithm. This algorithm can estimate the relative proportions of different cell types in a mixed cell population, which is crucial for understanding the immunological mechanisms of CD. We calculated the relative proportions of 22 different subtypes of immune cells (LM22) in the sample, which helps us understand which immune cells may play a key role in CD.

### 2.8. MR

We conducted MR analysis on the correlation between exposure and outcome variables in GWAS using the TwoSampleMR package in R. CXCL1 gene ebi-a-GCST90000458 was used as the exposure, and CD was used as the outcome finn-b-K11_CROHN (sample size ncase 2056; ncontrol 210,300, European), identifying any possible causal relationships. Given the scarcity of SNPs with genome-wide significance, we applied a more lenient threshold (*p* < 5 × 10^−6^) for the initial screening of SNPs. Nevertheless, we still found a significant association between gene expression and exposure related to CXCL1.To confirm the unique association between each SNP and risk factors, we used PLINK clustering method to evaluate linkage disequilibrium (LD). We established an SNPLD coefficient *r*^2^ > 0.001 and excluded SNPs < 10, 000 base pairs apart to ensure their independence and minimize the effects of pleiotropy. Strong statistical validation: We evaluated the strength of each IV using the *F*-statistic, defined as *F* = (*β*^2^)/SE^2^, where *β* represents the effect size of the allele and SE is the standard error. Exclude SNPs with *F*-values below 10 to avoid bias that may be caused by unmeasured confounding factors. We carefully selected SNPs related to instrument variables based on GWAS data related to CD. The “harmonic data” feature in TwoSampleMR is crucial for aligning allele directions between exposure and results, thereby eliminating any palindromes and incompatible SNPs. The data synthesis based on composite *p*-values mainly uses the inverse variance weighting (IVW) method. This method assumes the reliability of all IVs.

## 3. Results

### 3.1. Determination of DEG

**Figure 1:**
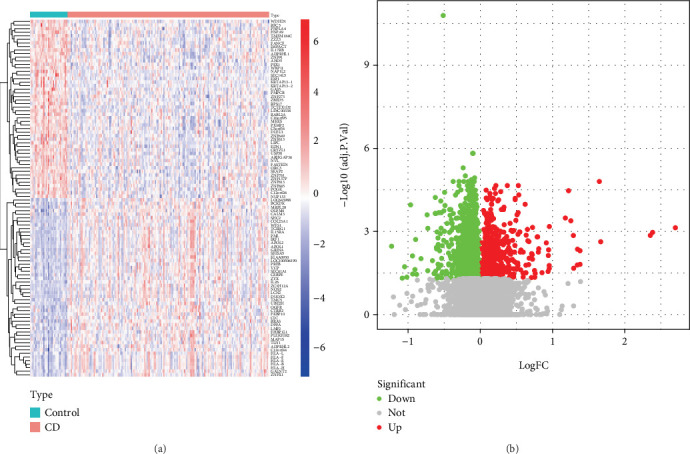
Visualization of DEGs in Crohn's disease. (a) Volcanic map and (b) heat map.

Using data on CD that we got from the GEO database, we performed differential expression analysis to find important DEGs related to CD. Visualization of DEGs is shown in Figure 1a,b.

### 3.2. WGCNA Analysis

**Figure 2:**
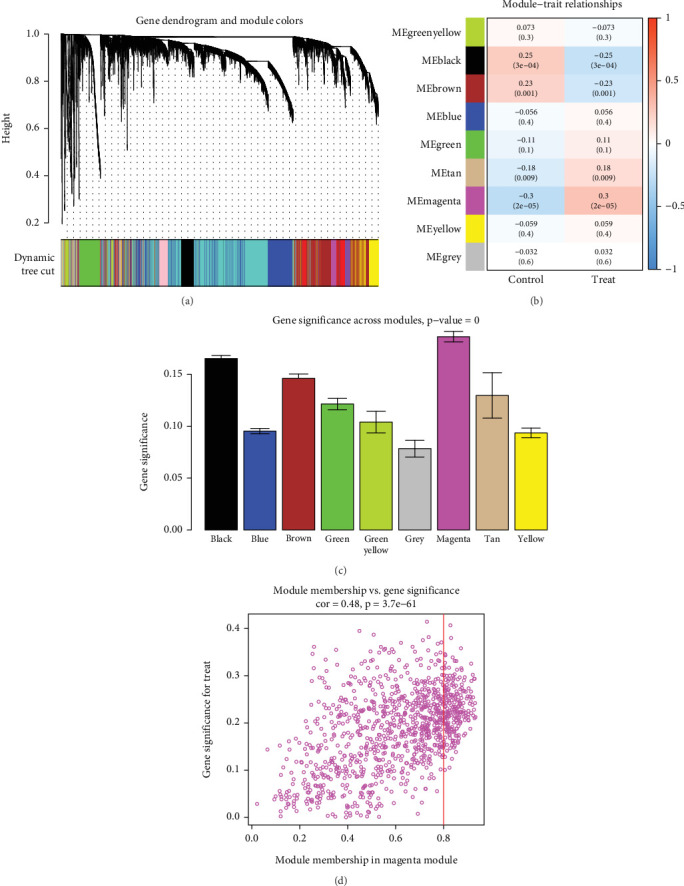
WGCNA analyzes important modules. (a) Cluster the dendrogram of genes. (b) The feature heatmap. (c) Positive correlation with CD in the scatter plot of the magenta module. (d) Module membership in magenta module.

WGCNA analysis was performed on the GSE179285 dataset to evaluate gene expression related to CD (Figure 2). Nine different module genes were identified (Figure 2). Through positive correlation coefficient analysis, the most significant correlation was ultimately determined for the magenta module (Figure 2).

### 3.3. Enrichment Analysis

**Figure 3:**
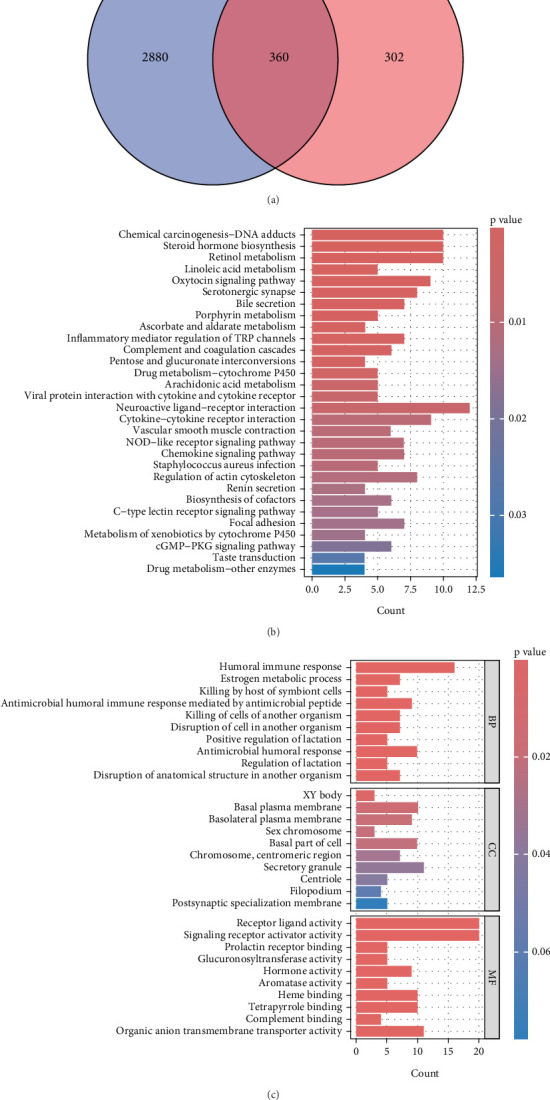
Screening of intersecting genes. (a) The Venn diagram of intersecting genes. (b) Pathway analysis of KEGG. (c) GO enrichment analysis.

The WGCNA method identified 360 intersecting genes between CD related core genes and DEGs. It is important in the progress and treatment of CD (Figure 3a). Perform GO and KEGG enrichment analysis on intersecting genes to explore their biological functions (Figure 3b,c). GO analysis showed that intersecting genes were significantly correlated with positive regulation of heme binding, tetrapyrrole binding, carboxylic acid binding, and organic acid binding. KEGG analysis showed that hub genes mainly affect the IL-17 signaling pathway and amoebic disease pathway.

### 3.4. PPI Network Analysis

**Figure 4:**
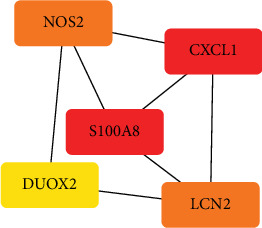
PPI network. Protein–protein interaction (PPI) network diagram of intersecting hub genes. The core gene map in protein interaction networks, where the depth of colors represents the score of genes.

The STRING online tool established a PPI network for intersecting genes (Figure 4a) and visualizes the Top 5 genes within the connection nodes (Figure 4b). The five hub genes include CXCL1, LCN2, NOS2, S100A8, and DUOX2.

### 3.5. Nomogram Construction

**Figure 5:**
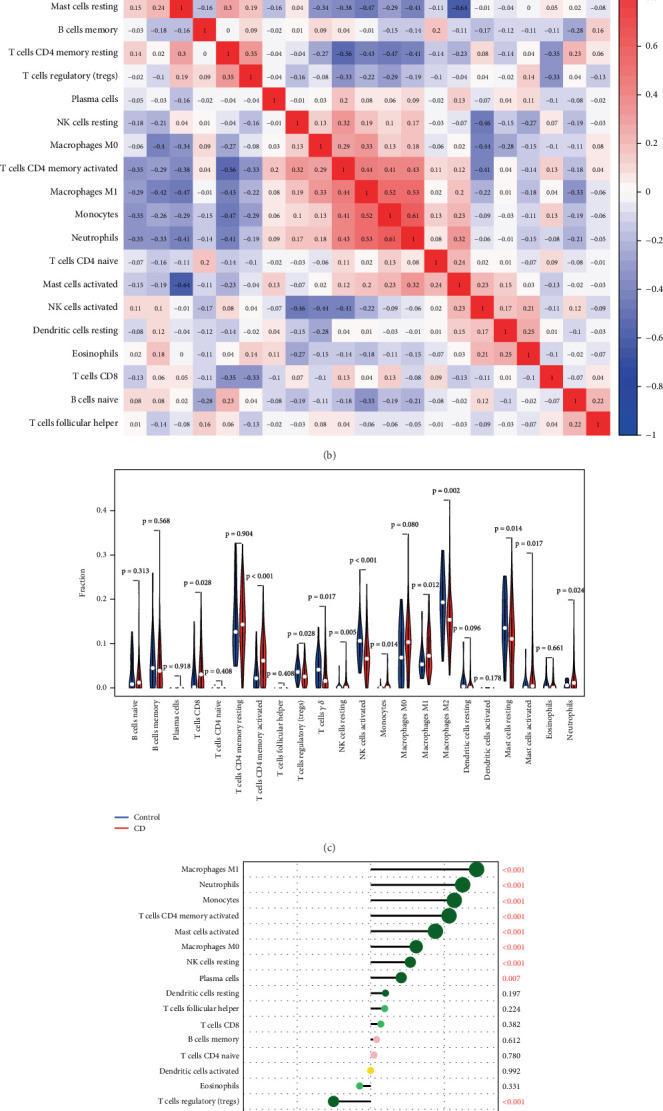
Nomogram model constructed for predicting the risk of Crohn's disease:(a) Nomogram model constructs based on key central genes (hub genes) to predict disease risk. (b) the predictive accuracy of the model by calibrating the curve to ensure its reliability. (c) CD through receiver operating characteristic (ROC) curves. (d) Lollipop diagram of correlation between CXCL1 and infiltration immune cells in CD. (e) Scatter plots of correlation between CXCL1 and infiltration immune cells in CD.

To forecast the risk of CD, we employed a nomogram model, as Figure 5 illustrates. Calibration curves were utilized to validate the predictive capabilities of the model, and the outcomes demonstrated that the model possesses a high degree of accuracy in determining the risk of CD, as illustrated in Figure 5. To assess the diagnostic performance of the five core genes, we also created ROC curves; as Figure 5 illustrates, an AUC of more than or equal to 0.7 was deemed clinically significant. The genes CXCL1, LCN2, NOS2, S100A8, and DUOX2 have AUC values of 0.831, 0.837, 0.786, 0.774, and 0.777, respectively, suggesting that they may be useful in the diagnosis of CD. Figure 5 displays the particular data.

### 3.6. Immune Cell Infiltration

**Figure 6:**
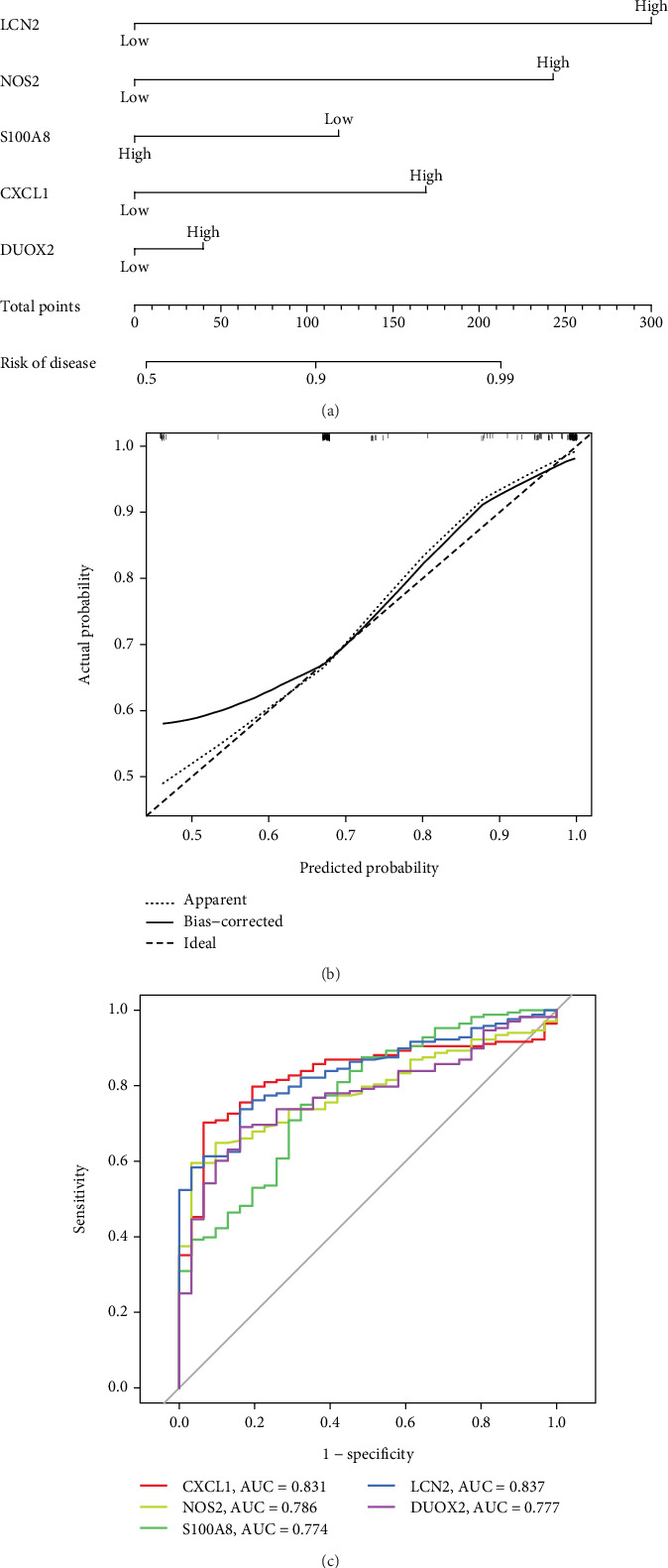
The investigation of CXCL1's immunological function in Crohn's disease (a) Presented information on the relative proportionate distribution of 22 distinct immune cell types. (b) The variations in immune cell infiltration in Crohn's disease. (c) Examined the relationship between immune cell infiltration and CXCL1.

The CIBERSORT algorithm was used to analyze the proportion of immune cell infiltration between the CD group and the control group (Figure 6a). The CD group showed a higher proportion of monocytes, macrophages M1, activated mast cells, neutrophils, NK cells resting, and T cell CD8. However, in the CD group, the infiltration ratio of CD4 memory–activated T cells, resting mast cells, mast cells, M2 macrophages, activated NK cells, dendritic cells, and Tergs was lower (Figure 6b). According to the correlation with 22 types of immune cells, CXCL1 is positively correlated with M1 macrophages (*r* = 0.72), monocytes (*r* = 0.57), NK cell activation (*r* = 0.48), mast cell activation (*r* = 0.44), CD4 memory resting T cells (*r* = 0.53), M0 (*r* = 0.31), and neutrophils (*r* = 0.62). On the contrary, CXCL1 was negatively correlated with M2 macrophages (*r* = −0.35) and Tregs (*r* = −0.25) (Figure 6c). Therefore, it can be inferred that immune cell infiltration is related to differences in gene expression.

### 3.7. CXCL1 and the CD

**Figure 7:**
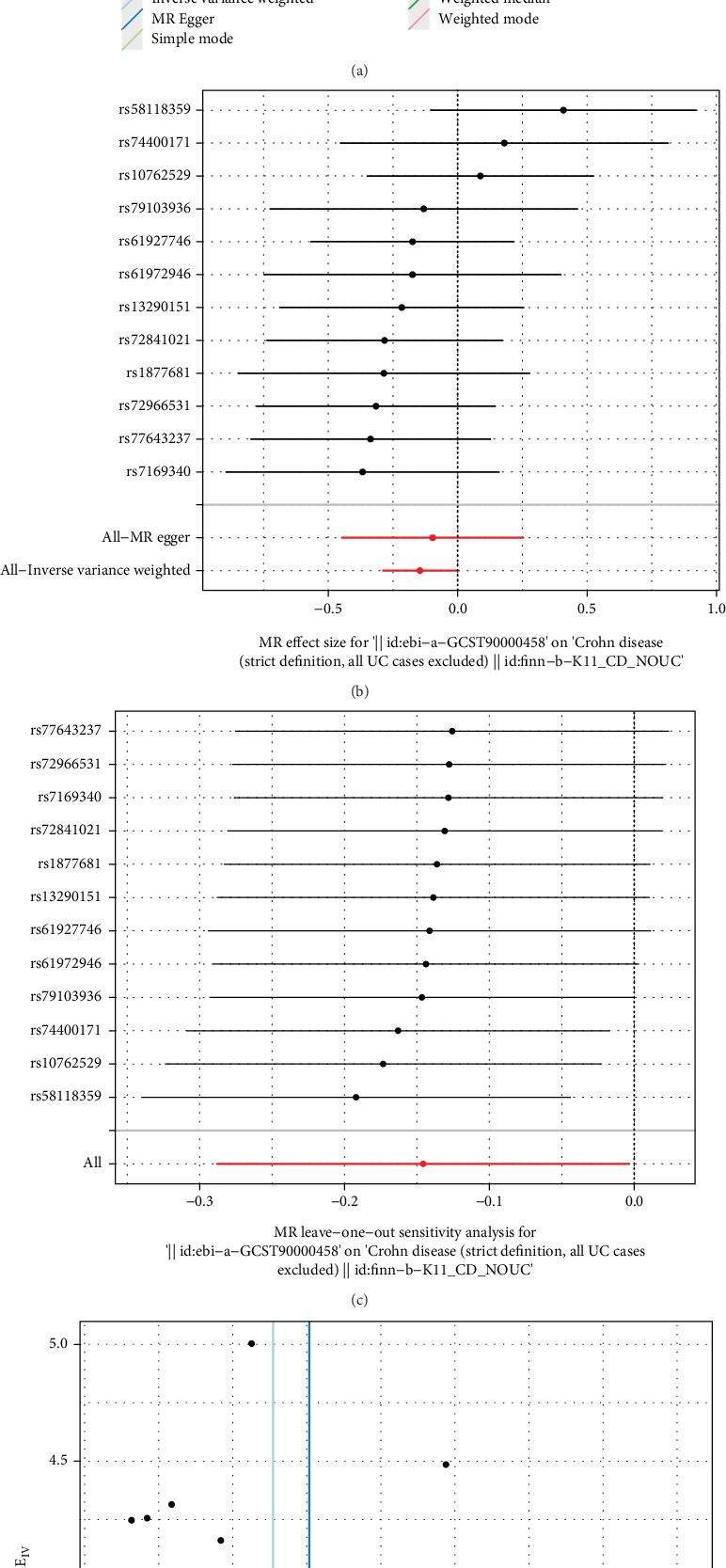
Results of Mendelian randomization study. (a) Scatter plot. (b) Forest plot. (c) Funnel plot. (d) Leave one plot.

MR analysis in GWAS can identify possible causal relationships between exposure and outcome variables. Given the scarcity of SNPs with genome-wide significance, all SNPs were checked in https://ldlink.nci.nih.gov/?tab=ldtrait. Specifically, we analyzed to adhere to rigorous statistical analysis and significance thresholds. This included the inclusion of sample size/power calculation, appropriate statistical tests, and strongly independent genetic instruments that are robustly associated with the risk factor of interest. While these thresholds can vary, we contained detailed information regarding the choice of *p* value cut-offs in methods section (*p* < 5 × 10^−6^). The IVW method is relatively straightforward to implement and computationally efficient. It involves weighting the estimates by the inverse of their variance, which allows for a quick and effective aggregation of information. This simplicity makes it particularly useful in contexts where computational resources are limited or when dealing with large datasets. In the context of MR studies, the IVW method is designed to estimate causal effects using multiple genetic variants as IVs. It is robust to certain violations of assumptions, such as horizontal pleiotropy, as long as the InSIDE (independent of the direct effect) condition is met. The IVW method provides clear and interpretable results in causal inference studies. The causal effect estimates are directly derived from the weighted average of individual IV estimates, making it easier to understand the relationship between exposure and outcome. The IVW method is particularly effective when multiple valid IVs are available. It leverages the information from all instruments to produce a more precise estimate of the causal effect. Using the IVW, we identified a significant association between CXCL1 and the risk of CD, with an OR of 0.864 (95% CI 0.979−0.984, *p* = 0.045) and a weighted median with an OR of 0.822 (95% CI 0.683−0.990, *p* = 0.039). MR-Egger intercept = −0.02, *p* = 0.77, There was no horizontal pleiotropy. We performed an assessment if selected genetic variants are associated with potential confounders thorough evaluation of the suitability of these SNPs based on published literature. They are strong IVs with *F* > 10. Additionally, considering the essential requirements of strong IV assumptions, we use R software to analyze and plot the causal relationship between CXCL1 and CD (Figure 7). The MR Egger test showed no heterogeneity in the results (Figure 7). The intercept results of the MR Egger regression for pleiotropy showed no pleiotropy, indicating the robustness of the data. In the sensitivity test where one item was omitted, no polymorphism was found, which confirms the reliability of the data analysis (Figure 7).

## 4. Discussion

CD presents a significant challenge in medical management [1, 4]. Recent advancements in genomic research have enabled a more nuanced understanding of CD, finding biomarkers through the quantification of gene expression levels [14, 17–21]. Early diagnosis is pivotal, with researchers actively engaged in the analysis and verification of CD-related biomarkers to pinpoint optimal treatment targets. The heterogeneity of CD underscores the importance of recognizing its dynamic and individualized pathology. Treatment strategies must be adaptable to the evolving condition of the patient. We use PPI analysis to identify five hub genes associated with CD, offering new avenues for therapeutic intervention [5]. This approach highlights the complexity of CD and the potential for personalized medicine in managing the disease. CXCL1 (chemokine ligand 1) is an important chemokine involved in the pathogenesis of various inflammatory diseases, including CD. CXCL1 is upregulated in inflammatory responses and promotes the chemotaxis and migration of neutrophils and other immune cells by activating the CXCR2 receptor. In the pathogenesis of CD, CXCL1 may function through the following biological mechanisms. The high expression of CXCL1 at sites of inflammation can promote the recruitment and activation of neutrophils, thereby exacerbating intestinal inflammation. This chemokine enhances the adhesion and migratory capacity of inflammatory cells through its interaction with CXCR2, leading to inflammatory damage to the intestinal mucosa. The expression of CXCL1 is regulated by various cytokines. IL-17, for example, increases the stability of CXCL1 mRNA by activating the NF-*κ*B pathway and the MAPK p38 signaling pathway, thereby promoting its expression. This regulatory mechanism allows CXCL1 to continue functioning in the inflammatory microenvironment, maintaining and amplifying the inflammatory response. CXCL1 may modulate the interaction between intestinal epithelial cells and immune cells, thereby affecting the integrity of the intestinal barrier and the homeostasis of the microbiota. CXCL1 promotes the proliferation and migration of inflammatory cells by activating the NF-*κ*B/P300 signaling pathway. Additionally, CXCL1 may influence the intensity and duration of the inflammatory response by affecting other signaling pathways, such as the MAPK pathway.

In the quest to unravel the complexities of CD, our study employs a comprehensive bioinformatics approach, notably the WGCNA analysis, to identify key genes that serve as potential hubs in the pathogenesis of the disease. Through this analysis, we have pinpointed CXCL1, LCN2, NOS2, S100A8, and DUOX2 as candidate genes. We performed a thorough assessment of the diagnostic model's prediction power and showed that it is sensitive and specific in predicting the risk of developing CD. The calculation of AUC values for these genes, along with the construction of an ROC curve, further solidified the diagnostic efficacy of our approach. The research also underscores the significance of cytokine regulation in CD, suggesting that these molecules may act as core genes.

The IL-17 signaling pathway, known for its role in inducing proinflammatory transcription factors through NF-*κ*B, MAPK, and C/EBP cascades [22–25]. As single-gene products, cytokines are regarded as important mediators of immunological responses and inflammation. They primarily function in different ways in CD by binding to cell membrane receptors and starting particular intracellular signaling cascades [26]. As a result, transcription factors are activated and cellular processes are regulated, which makes cytokines a more attractive therapeutic target in the CD sector. As a crucial mediator in the inflammatory response, CXCL1 has been found to activate neutrophils when they attach to immune complexes and may potentially inflict ischemia injury on surrounding tissues. CXCL1 is now a viable candidate for a cutting-edge therapeutic intervention for CD as a result of this study. As such, this work establishes the groundwork for subsequent investigations and clinical implementations targeted at enhancing CD treatment.

In the colon tissue of individuals with CD, we found a strong relationship between particular immune cell subtypes and biological processes using CIBERSORT analysis. In particular, there has been an increase in the quantity of neutrophils, M1 macrophages, active mast cells, monocytes, resting NK cells, and CD8 + T cells. Concurrently, there was a decline in the quantity of resting mast cells, gamma delta T cells, active NK cells, dendritic cells, regulatory T cells (Tregs), activated memory CD4 + T cells, and M2 macrophages. Furthermore, we discovered a connection between neutrophil infiltration in colon tissue and CXCL1. This study does, however, also have certain difficulties. First off, because the study is retrospective in nature, it is challenging for us to quickly get comprehensive clinical data, such as patients' treatment status at the time of data collection. This makes it more difficult for us to thoroughly identify disease-specific subtype biomarkers. Second, this investigation included inflammatory tissue samples from the colon and ileum regions, although some ileum-specific diagnostic genes were not fully confirmed due to data limitations imposed by the GEO database. Therefore, in order to more fully elucidate the process of immune cell infiltration in CD, future research must include prospective designs. Our research showed the critical role of CXCL1 as a hub gene in CD, highlighting its potential as a novel therapeutic target. Utilizing Cytoscape software analysis, CXCL1 emerged as a significant hub gene within the PPI network, underscoring its centrality in mediating hyperimmunity in IBD. This aligns with findings by Zhu et al., who demonstrated that tungstate can manipulate the microbiota to suppress intestinal inflammation, concurrently inhibiting the expression of CXCL1 [27]. As a chemotactic factor for neutrophils and a ligand for CXCR2, CXCL1 is instrumental in the recruitment of appropriate immune cells in CD [28–30]. S100A8, a calcium-binding protein predominantly found in neutrophils, is another key player identified in our study [31]. S100A8 induces cytokine secretion from peripheral blood mononuclear cells, thereby amplifying the inflammatory response. In IBD, particularly in autoimmune diseases like CD, S100A8 is present in high concentrations and is released to stimulate cytokine secretion [30, 32]. Lipocalin-2 (LCN2), an inhibitory protein involved in inflammation regulation and metabolic homeostasis, upregulated in the serum of patients with CD [26]. Its elevated levels suggest potential biomarker for CD [26, 33, 34]. Furthermore, LCN2's upregulation in CD may be associated with a protective effect [35, 36]. The interaction between NOD2 variants and DUOX2 has been implicated in the inhibition IL-10 transcription [37, 38]. It is known that DUOX2 is an essential host factor for preserving intestinal integrity, and that detrimental mutations in this gene may occur before the symptoms of IBD appear [37, 39, 40]. The findings support the work in [41], which highlights the importance of DUOX2 in intestinal flora abundance and their regulatory roles in enterocyte function and innate and adaptive immune responses. Specifically, our data show that DUOX2 expression is upregulated in the ileum and colon of CD patients. In conclusion, our research offers a thorough examination of the major genes and proteins connected to CD, providing fresh perspectives on possible treatment targets and biomarkers for diagnosis. The results highlight the intricacy of CD pathophysiology and the promise of personalized medicine techniques for its treatment.

The association between CXCL1 levels and the risk of CD was examined using the dual sample MR approach. The findings suggest a link between CXCL1 expression and CD risk. By avoiding systematic biases such reverse causal effects and confounding variables that can taint the results of observational research, multiple regression analysis (MR) increases the trustworthiness of the findings [42]. Only demographic data from Europe was used in the study in order to reduce the influence of confounding variables. There is no data sensitivity nor horizontal pleiotropy, according to the findings of the MR Egger and omission sensitivity analyses. There are various restrictions on the research. First off, we can only utilize one dataset because the CD dataset is not readily available in the GEO database, which might compromise the accuracy of the results due to the small sample size. Second, only bioinformatics techniques were used in this work to examine genes and possible functions they may play in CD [42]. The reliance on a single dataset (GSE179285) with limited sample size raises concerns about generalizability. Future validation in larger, multicentric cohorts is still needed. Additional research is required to confirm and broaden our conclusions.

CXCL1 is an emerging inflammatory biomarker that has shown unique potential in the study of IBD. Compared with traditional biomarkers such as fecal calprotectin, the expression level of CXCL1 in ulcerative colitis (UC) is closely related to the infiltration of various immune cells, including neutrophils, M1 macrophages, and activated dendritic cells. This correlation suggests that CXCL1 may have higher sensitivity in detecting inflammatory activity. CXCL1 promotes the chemotaxis and migration of neutrophils by activating the CXCR2 receptor, thereby exacerbating intestinal inflammation. This mechanism is different from that of calprotectin, which is mainly a protein released by neutrophils and is used to detect the presence of intestinal inflammation. The high expression level of CXCL1 in UC is closely related to the degree of inflammatory activity. In contrast, although calprotectin increases during active inflammation, it has limitations in distinguishing mild activity between CD and UC. Therefore, CXCL1 may have an advantage in monitoring the disease activity of UC.

## 5. Conclusions

We established a coexpression network using WGCNA and identified hub genes associated with CD. The study screened five potential biomarker genes in CD patients using a bioinformatics approach and MR study. Our results provided insights into CXCL1, LCN2, NOS2, S100A8, and DUOX2 in CD and suggested that CXCL1 may potentially be an optimal biomarker that could be a relatively easy path to clinical translation. Furthermore, it provides a certain reference value for further exploring the role of these biomarkers in CD diagnosis and treatment.

## Data Availability

The authors have nothing to report.
